# Measurement in the Ultimatum and Dictator Games: Construct validity, standardization and the problem of coordination

**DOI:** 10.1007/s40656-026-00725-6

**Published:** 2026-09-16

**Authors:** María Jiménez-Buedo

**Affiliations:** Department of Logic, History and Philosophy of Science, UNED (Universidad Nacional de Educación a Distancia), Paseo de Senda del Rey 7, 28040 Madrid, Spain

**Keywords:** Problem of coordination, Construct validity, Dictator game, Ultimatum game, Measurement

## Abstract

The ultimatum game (UG) and the dictator game (DG) are two of the most prominent tools in experimental and behavioural social sciences. Originally designed to study bargaining behaviour and decision-making in economics, these simple yet versatile scripts have come to be viewed by many as reliable instruments for measuring prosocial preferences, such as altruism, generosity, or sensitivity to social norms. Proponents of the measurement view have argued that the UG and DG serve as standardized “social thermometers,” capable of revealing cultural and contextual differences in normative behaviour across diverse populations. For instance, the UG has been used to demonstrate the influence of institutional contexts and cultural norms on fairness expectations, while the DG has been described as a straightforward measure of altruism. In this paper, we critically examine the idea that the UG and DG function as robust measuring devices, raising key questions about their validity as tools of measurement. We argue that the constructs these games are purported to measure—ranging from prosociality to norm compliance—are often imprecisely defined and vary across studies, and this severely limits the sense in which the DG or the UG can be interpreted as measuring devices. By analysing the ontological and methodological challenges surrounding these games, we contend that the UG and DG are better understood as tools for exploratory experimentation rather than as reliable instruments for systematic measurement.

## Introduction

The new experimentalism in the social sciences has been greatly influenced by games or scripts that were initially created by experimental and behavioural economists and that are currently employed by a broader social scientific community of researchers. Many of these games are well known among social scientists and even among the more general public because of their results, which sometimes can defy our intuitions about rationality, social desirability or generosity.

The ultimatum game (UG) and the dictator games (DG), both characterized by their very simple structure, are among the most popular games, not only in Experimental and Behavioural Economics, but also, in the broader new experimental Social Sciences. In both cases, participants redistribute their money to other experimental subjects in ways that depart from economic rationality narrowly defined as payoff maximization.^1^

Although initially the results in these games were interpreted and invoked by some economists as displays of irrational behaviour (why should rational subjects voluntarily share their earnings with a fellow player whom they have just met?), these same results soon began to be interpreted as evidence of the existence and robustness of altruistic or (more generally) prosocial preferences. Their current uses have been linked to the *measurement* of these prosocial preferences, so for example Hilbig et al. (2015) say of the DG that is “commonly considered the most straightforward measure of altruistic behaviour”, and the UG has been compared (notably, by Guala, 2008), to a “social thermometer” that measures SENSITIVITY to social norms.

Take the ultimatum game and Guala’s reconstruction of its reception and uses among experimental social sciences: the success of the UG (or as Guala says, what makes it a “paradigmatic experiment”) is explained precisely, by its versatility. Developed in the 1980s to study bargaining behaviour, the standard ultimatum game (UG) results were soon conceived as a test of economic theory, in that they defied traditional game theory predictions, showing that people tend to reject unfair offers instead of opting for maximizing revenue. For this reason, these results were interpreted as a challenge to the standard assumptions of self-interested rationality. Guala describes how the game’s initial success lays precisely in its capacity to reveal nonstandard preferences, particularly regarding fairness. Yet, once these results become part of the shared background of experimentalists, the UG’s job transforms into its ultimate role: its current function is that of serving as a *measurement device*.

According to Guala, the UG acquired this role first via a series of experiments by Alvin Roth and his collaborators (1991), that made systematic comparisons between the UG and a market exchanges ruled by competition, thus inaugurating the role of the UG has a means to test the idea that institutions matter, or that the institutional framing of a given situation can shape the outcomes and filter the participants perceptions regarding what is a fair outcome.

This line of research was famously taken up by a multidisciplinary team of researchers led by the anthropologist Joseph Henrich. In a massive feat of comparative work, the team delivered a series of studies across 15 small-scale societies in South America, Africa, and Asia, in which they had groups of people in each of these communities play several economic games such as the UG, the DG, and the Public Goods Game, in an attempt to understand the role of culture in shaping economic decision-making (Henrich & Smith, 2004). A few years earlier, Henrich’s research had revealed surprising variations in behaviour in the ultimatum game (UG) among the Machiguenga people of Peru, who, unlike Western subjects, proposed more unequal splits and rejected unfair offers less frequently (Henrich, 2000). Henrich had explained these results through detailed ethnographic data regarding how the Machiguenga interpreted the UG based on their more limited exposure to market-like institutions. The finding then was interpreted to support the idea that cultural norms significantly influence behaviour, leading to a diverse range of fairness expectations. These different normative expectations were then reflected in the different UG average results in different populations.

By the mid-2000s, the use of scripts such as the DG and the UG to test hypotheses regarding the effects of different normative cues was well embedded in the practices of experimental social scientists. Guala would describe this, for the UG, as a case where the script was being used as a *measurement* stick—a social thermometer. But in what sense, then, was the UG a social thermometer?

According to Guala, if we have different pools of subjects from different cultural backgrounds, we can use the UG as means to test their sensitivity to different normative cues: drawing parallels with Chang’s account (2004) of the standardization and coordination involved in the coming of the concept of temperature, Guala describes the UG as a *portable measuring device*, same as a standardized, portable thermometer that we carry around different locations if we for example want to test, whether “water boils at different temperatures at sea level and on a high plateau”.

Although Guala’s articulation of the idea is more explicit in this regard than most authors, the idea that the DG and UG can act as *measuring devices* is well embedded in the relevant literature and among experimentalists themselves, and examples abound with explicit references to this avail.^2^ Although the number of references that mention either game as a *measurement tool* is immense, we can perhaps single out as particularly relevant the statements of two central figures in behavioural Economics, Fehr and Camerer who claimed that “[r]egardless of which models are most accurate, psychologically plausible, and technically useful, the important point for social scientists” these games “can be used to *measure* social preferences” (2004, p. 84).

In this article, we take issue with the idea that the UG and the DG can act as measuring devices and ask ourselves in what sense they can be deemed so. If these scripts (UG and DG) can actually act as measuring devices, what do they measure? Given that they are used in different experiments to represent different concepts (such as altruism or norm sensitivity), we should ask ourselves: in virtue of what properties would these scripts earn that type of versatility? Can the same measuring device be used to measure different constructs^3^ or is there instead a broad, encompassing construct (such as “social preferences”) that can be meaningfully invoked to say that this is what these games measure? The imprecise and fuzzy nature of this and related notions, such as prosociality, invites yet another critical question: in what sense can there be measurement when the construct representing the measurand is left to be only loosely defined? In this paper, we address these questions by qualifying the limited sense in which the UG and DG can be regarded as measurement instruments.

In the first section, we examine some examples of experimental practices that assume that the ultimatum and dictator games can be said to measure one (and the same) underlying construct—a claim often made by both practitioners and commentators, that assume or maintain that these games measure prosociality. We then introduce some of the central ideas to the new philosophy of measurement, with particular attention to two concepts. The first is the *problem of coordination*, understood as the challenge to connecting a measurement procedure to the concept or construct that it is intended to measure. The second is the the notion of *epistemic iteration*, which emphasizes that progress in both the measurement of a phenomenon and its conceptual definition often occurs through an iterative process, in which refinements in measurement practices and conceptual understanding mutually inform and shape each other. Framing our discussion in these terms reveals significant differences between paradigmatic measurement practices and the kinds of experimental exercises exemplified by the ultimatum and dictator games.

We then show how, given the variety of experimental practices surrounding the DG and UG, there is not a meaningful way to in which we can assert that there exists a single entity or property that each of these games measure, nor that measurement is the primary epistemic function of these experiments. In the following section, we identify two dominant uses of these games in experimental social science:

1. As *situational analyses*, designed to explore how individuals behave under specific institutional or strategic conditions.
2. As *population analyses*, aimed at comparing behavioral tendencies across groups or cultures.

We then discuss the conceptual and methodological problems that arise when these two experimental games are interpreted as *measurement devices* in either of these contexts.

We show that both games are primarily employed as tools for hypothesis testing, rather than to measure an often undefined construct. We also explain how the lack of clear construct definition helps explain why the results of these experiments are best understood in local, context-specific terms.

We then examine the interpretation of these games as instruments for measuring norm sensitivity. Returning to the example of the Machiguenga’s UG results, we argue that the game fails to address the problem of measurement standardization, i.e., the challenge of ensuring that measurements obtained across different contexts are comparable and reliable through adherence to shared, established standards.

Finally, we conclude by emphasizing the continuing value of the UG and DG as tools for hypothesis testing, while cautioning against treating them as genuine instruments of measurement.

## What does it mean for these games to *measure* social preferences?

Experimental economics was initially less concerned with measurement than with hypothesis testing and with the controlled reproduction of market phenomena. Early experimental work in Economics in the 1970s and 80s—such as Vernon Smith’s market experiments—aimed primarily at demonstrating how market institutions could emerge and function under laboratory conditions, rather than at quantifying specific psychological or behavioral constructs. In contrast to much of psychology, where experimentation was early on tied to the measurement of mental processes or sensations, experimental economics only later developed an interest in something that some contemporary practitioners have come to identify as the “measurement” of preferences. This shift came with the rise of behavioral and experimental approaches that explored so-called *social preferences*—such as fairness, trust and reciprocity—through games like the ultimatum, dictator, and trust or public good games.

By the mid 1990s, and under the auspices of the MacArthur Foundation, a group of economists, dissatisfied with the standard Economics treatment of agents’ preferences, created an interdisciplinary group that was to be known as the MacArthur Preference Network. The need to create this research group came from the view that Economics was limiting its applicability and validity by excessively focusing on preferences that were ‘self-regarding’ and ‘outcome oriented’. Scholars in the Preference Network thought that this view of human nature, which they referred to as the *selfishness axiom*, was too limited. In this context, the laboratory began to appear to these practitioners not only as a setting for testing theoretical predictions, but also as a site where *social preferences* could be observed, inferred, and even measured. Two of the leading figures in the group, when talking about three paradigmatic games (UG, the DG and the Public Goods game), share this vision when they say that “[i]n addition to *measuring social preferences and social norms* [these] experimental games may also be used for *measuring* moral authority, players beliefs about other players’ actions in coordination games, cultural homogeneity, and status effects in bargaining” (Camerer & Fehr, 2004, p. 92).

Partly in virtue of its simplicity, the DG soon became, much like the UG, a ready tool to explore different aspects of prosociality by two different means: the comparison of results *across populations* (do students give more or less than workers, do women give more or less than men?) and a second route, related to *situational factors* (e.g., do people give more if the Recipient is anonymous, or if he is she is identified?, do people give more to Recipients after reading a statement promoting certain values?).

Engel’s meta-analysis of the dictator game, published in 2011 (Engel, 2011), stands as an interesting and widely referenced piece in that it constitutes an early attempt to synthesize findings from various studies that employ the DG script in their hypothesis testing. This meta-analysis regresses the results of different DG variations on the quantity that Dictators tend to give. The latter variable is often referred to by Engel in his meta-analysis as a parameter that reflects “generosity”, a terminological choice that is common among experimentalists to refer to the variable measuring donation levels in the DG.

Since Engel published his meta-study, the quantity of treatments and variations implemented in new DG exercises has at least cent-fold, as social scientific labs, where the DG script is a staple, have proliferated across universities worldwide due to the explosion of experimental practices in the social sciences. The picture drawn in Engel’s exercise (based on a sample of 445 studies) already showed in 2011 some patterns that drew the interest of many researchers and further stimulated research. For instance: Engels’ meta-analysis revealed that women typically exhibited greater generosity than men as Dictators, received less as Recipients, and were generally expected to give more. Furthermore, older individuals tended to give more than younger ones, while in the rare cases in which children acted as experimental subjects, very few of them gave nothing. Regarding studies delving into “situational factors,” and how they affected the level of allocations, we can also talk of certain patterns, even if the synthesis of results poses more challenges. To mention just a couple of them: lower stakes in the game correlated with increased amounts transferred to Recipients (or put differently, Dictators tend to donate less the higher the stakes). Also, evidence indicated that the identity of the recipient often influenced allocations, with those recipients perceived as more deserving receiving a larger share.

We should pause here for a moment to reflect on what is behind Engel’s exercise, his terminological choice, and the assumptions lying behind the idea that the DG’s results can be aggregated together in a meta-analysis. A meta-analysis of DG results assumes that all these experiments measure varying amounts of the same underlying phenomenon. Engel refers to this variable—the level of dictator donations—as “generosity.” Combining data from diverse DG variants thus implies that each variant or version of the DG yields different *measures of the same measurand*.

But what is this measurand, then, and how should we understand this (loosely defined) concept of “generosity? And what is the theoretical foundation for terminological choice? Unfortunately, Engel’s meta-analysis is in this sense representative of a relevant proportion of the literature employing the DG or UG as scripts, offering no theoretical account or explication of the variable representing allocation levels—or what Engel terms *generosity*. From the perspective of the theory and philosophy of measurement this represents a puzzling stance, for without a theoretical clarification or operational grounding of a construct, its status as a measurand remains conceptually opaque. In this sense, in the theoretical and philosophical accounts of measurement, measuring and conceptualizing go hand in hand: there cannot be an attempt to measure a construct if there is not an attempt at conceptualizing it.

Many researchers however employ the dictator game (DG) and ultimatum game (UG) without engaging in theoretical or conceptual discussions about what these games are meant to represent. Specifically, they often overlook the nature of the construct behind the variable typically used—namely, the level of donations or allocations. This omission could suggest that an operational strategy to find a definition is implicitly accepted: generosity, or prosociality, is defined simply as whatever the DG or UG measures.

Such an approach might be interpreted charitably as an instance of exploratory, inductive concept formation. From this perspective, researchers are not presupposing a fully developed concept of generosity or social preferences but are instead using these experimental tools to help discover and refine one. This seems to be the spirit of early interpretations by scholars like Camerer and Fehr, who framed these games as measuring tools in the context of what they thought, somewhat 20 years ago, was a nascent theory of social preferences, to be built against the backdrop of what they thought was a too restrictive, limiting view of preferences (often conceived solely as self-regarding preferences). In this view, the DG and UG could be seen as part of a broader attempt to build, inductively, a theory of prosocial behavior through empirical patterns.

As mentioned, if we adopt this inductive reading, we can understand the widespread use of these games without conceptual elaboration as a kind of provisional operationalism: we might not feel that we need to define generosity because we commit to the idea that generosity is defined as whatever is measured by the DG. This definition would be undeniably circular (the DG measures generosity, yet generosity is that which is measured by the DG), but does this need to be a problem? According to the new philosophy of measurement, a certain degree of circularity in measurement is not only not problematic, but rather necessary. However, we see next how this circularity in measurement needs to be accompanied by sustained conceptual effort.

## Measurement as a gradual achievement: epistemic iteration and the problem of coordination in the new philosophy of measurement

Philosophers such as Chang (2004) and Tal (2020) have shown that circularity in the early stages of measurement development can be productive rather than problematic. The history of thermometry provides a helpful example: the concept of temperature and the instruments used to measure it were developed together in an iterative process. The concept of *epistemic iteration* describes how the grasping of a phenomenon progresses through a continuous process of refinement rather than a single leap. In this view, measurement standards and concepts evolve by repeated cycles of improvement, where each stage builds upon and corrects the limitations of previous ones.

In this way, Chang’s historical account of the advances of temperature measurement describes how early, imprecise thermoscopes led to the development of more reliable thermometers through iterative adjustments to scales, materials, and definitions (2004). This process directly relates to the *problem of coordination*, which concerns how abstract theoretical quantities (like temperature or mass) are reliably linked—or *coordinated*—to empirical operations and instruments. Epistemic iteration provides a pragmatic solution: coordination is not achieved once and for all but continually re-established as theory and practice co-evolve. Thus, the stability and objectivity of measurement emerge not from fixed foundations but from the success of an ongoing, self-correcting iterative process.^4^

Can we think of the DG and UG in a similar way, as early steps in the iterative development of a robust concept of prosociality or social preferences? Unlike the case of thermometry, we lack the benefit of hindsight to judge whether current experimental practices will eventually converge toward a stable and meaningful construct (Luchetti, 2024). However, as we try to demonstrate in what follows, there are reasons to be skeptical.

As we see next, it is doubtful that these experiments collectively constitute a coordinated effort to define a unified construct. As we show, the experimental practices around the DG and the UG suggest that researchers working with these scripts are not collectively aligned toward such an endeavor and a relevant proportion of experimental works conducting UG and DG studies are unconcerned with conceptual issues regarding the construct represented in the variable that measures the level of donations, since they use these games for hypothesis testing about other phenomena (such as discrimination).

The widespread use of these games across diverse research domains—addressing not only themes such as prosociality, and norm sensitivity, but also topics such as sexism or polarization, contributes to the absence of a cohesive conceptual effort to define what the experiments are actually measuring in each context. Instead, researchers often regard them as a flexible tool for investigating something as broad and undefined as “human sociality” (Engel, 2011).

### Measurement in the situational variants

As briefly mentioned above, in situational variants of the dictator game (DG) and the ultimatum game (UG), the baseline game serves as a control condition that is compared to a modified treatment. For simplicity, in this section we focus on the DG, since, being a degenerate form of the UG, it has a simpler structure that lends itself more easily to analysis. The modified treatment in the DG represents the intervention of interest: typically, this involves adding an extra element to the standard DG. For example, a standard DG played against an anonymous recipient may be compared to a modified version in which the recipient is, say, an NGO devoted to environmental causes.^5^ In this type of experiment, the results from the baseline and treatment groups are compared to identify systematic differences in the behavior of participants across the two conditions. In this type of experiment, results from the baseline and treatment groups are compared in order to identify systematic behavioral differences. Any observed variation in behavior is causally attributed to the additional cue introduced in the modified DG (Table 1). A classic example compares participants in a standard DG with those in a version where a visual cue (a picture of eyes on the wall) is introduced to create the impression of being observed. Results from the treatment group (the “eyes” condition) show higher allocations compared to the control group, suggesting that even an implicit reminder of being watched can increase the level of donations (Haley & Fessler, 2005).

**Table 1:** The situational variant of the DG

	Putative cause	Putative effect	All other factors
Experimental group (T) (a modified DG)	X	Y1	Constant
Control group (C) (A standard DG)	0	Y2	Constant

Causal effect of X = Y2 − Y1

As these examples make clear, the primary objective of this type of experiment is to test hypotheses regarding the effects of specific elements, such as cues or contextual factors, on the behaviour of Dictators in distributing allocations between themselves and a Recipient. This process of hypothesis testing follows the fundamental principle of experimental inference: if there is a statistically significant difference in means between the control and treatment groups, the experimenter can infer that the introduced element in the treatment condition has a causal effect on allocation levels.

The main purpose of these experimental exercises is thus to provide the basis for hypotheses testing, but could these experiments also be considered an exercise in measurement? In a trivial sense, there is certainly measurement going on. The experiment does *measure* the level of allocations in both the control and treatment conditions, just like any other experiment (or any type of hypothesis testing) would do. Yet, these measurements are a mere means to a determining whether the proposed treatment (i.e., the additional element introduced in the modified game) is causally efficacious. For the experimenter, the question of interest in this type of situational analysis is whether the treatment has a causal effect on allocation levels.

The different allocation levels represent distinct values of a variable, but this alone is insufficient to regard the experiment as an instance of *measurement* in the stricter, technical sense of the term. It is not measurement as conceived in model-based accounts—that is, as the coherent assignment of numerical values to parameters within a theoretical model of a process—nor in constructivist–operationalist terms, since these experiments do not contribute to a broader conceptual effort aimed at refining or stabilizing a given construct. Measurement thus conceived describes a process in which developments in the measurement procedure and conceptual refinements of the measurand go hand in hand: they progress iteratively when advances in the one produce changes in the other and vice versa. That is, we refine our constructs through our attempts of measuring them and, in turn, we improve in our measurement procedures of those constructs because of we refine them conceptually and this allows for improvements in their operationalization. By contrast, in the situational experiments we are now analysing, the testing of a causal hypothesis can proceed without a definite commitment to what, exactly, the two arms of the experiment are measuring, i.e. without a clear construct corresponding to the numerical values representing allocation levels. It suffices that we conclude that the putative cause we are introducing causally affects (or not) the variable representing the level of allocations.

This is clearly illustrated by Haley and Fessler’s (2005) study. While their results can, in a loose sense, be read as informing our understanding of *prosociality* or *generosity*, the authors’ primary aim is not to refine such constructs but to expand the methodological repertoire of the dictator game (DG). In this way, Haley and Fessler motivate and discuss their contribution as one that introduces *subtle cues that can alter behavior in the DG*, in contrast to other laboratory economic experiments that “focus on the effects of changes in the formal rules and parameters of the economic interactions at issue, with little attention paid to the cues of observability provided by the environment in which these interactions occur.” Their contribution thus lies in showing that subtle environmental cues can influence allocation behaviour, adding a novel dimension to the DG literature.

In this respect, the study operates independently of any specific theoretical commitment to what the allocation variable measures, whether *prosociality*, *norm compliance*, *altruism*, or *generosity*. At most, the findings might indirectly inform a broader discussion of *social preferences*, but this is not their direct purpose. The value of the work lies instead in illustrating a new experimental manipulation within the DG experimentation (one compatible with multiple interpretations of prosocial behavior), without seeking to adjudicate among them.

Something similar can be said, even more clearly, of other well-known examples in the literature that use the DG to test hypotheses that are unrelated to prosociality. Take, for instance, Whitt and Wilson’s classic (2007) article, in which dictators are found to give higher allocations to co-ethnics than to members of other ethnic groups in postwar Bosnia. Here, the authors effectively use the DG script to study how contextual variables—such as recent conflict—and attitudinal differences toward in-groups and out-groups can explain discriminatory behavior, whether positive or negative, in how agents treat individuals from different ethnic backgrounds corresponding to the former Bosnian war factions. The authors interpret the DG not only as a way of representing “perceptions of fairness,” but also as capturing “tastes for discrimination.”

In this case, the authors interpret that the DG is deployed as a measuring device *not* for prosociality, but for ethnic bias.^6^ The experimental variation between treatments—altering the ethnic identity of recipients—functions as an empirical marker of discrimination: how people behave differently toward others solely based on perceived ethnic identity. Yet, while the results are clearly relevant, they cannot be said to contribute meaningfully to the conceptual development of *prosociality* or to the refinement of a theory of *social preferences*. Rather, they offer a valuable empirical contribution to the literature on political attitudes in conflict-affected regions. The authors emphasize the practical significance of using behavioral measures for *detecting ethnic bias*. They argue that the DG experiment is valuable precisely because it allows researchers to observe *costly choices*, rather than relying solely on *attitudinal measures*, which may be unreliable: “Only relying on attitudinal measures may be misleading in that it costs people very little to reply to an abstract question about how others should be treated.” In contrast, their version of the DG forces participants to make real decisions with material consequences.

There is, of course, room for differing views on how these studies relate to broader theoretical developments on prosociality. One can consider that all instances of the DG or the UG, by dealing directly or indirectly with prosociality, contribute, even in some minute way, to our knowledge of social preferences. However, this does not imply that every instance of the DG or the UG constitutes an attempt at measuring or refining the concept of social preferences. While the studies in our examples do contribute valuable behavioral evidence relevant to understanding important topics such as discrimination, perceptions of fairness in postwar areas, or the causal effectiveness of subtle cues, they cannot be said to substantially engage with, refine, or extend the conceptual apparatus surrounding social preferences. Treating every empirical variation of the DG as a theoretical contribution to this endeavor risks conflating the collection of interesting behavioral data with genuine theory construction.

### Measurement “across populations”

The DG and the UG have not only been employed in situational analyses, but they have also been used extensively to identify differences in the mean allocations between populations. For example, as mentioned above, we now know that donations in the DG tend to increase with the age of the Dictator; that women tend to give more than men, or that student participants tend to give less than participants that are recruited in the general population (Engel, 2011). We also know that differences in UG behaviour are slim if we compare urban areas in developed economies amongst themselves, so players in Ljubljana play relatively similar to players in Tel Aviv, yet, important differences exist between those results and the behaviour of players in some areas of the world that are not characterized by full marketization of their economic system (such as some of the small-scale societies studied by the MacArthur Foundation project (Henrich & Smith, 2004).

The causal interpretation of this type of exercise is perhaps less straightforward than in the case of the situational analysis. In the case of population studies, the difference in behaviour among groups, if found, is interpreted as the identification of a property of the members of the group in relation to the way in which they play the game in question. Again, the question arises as to whether this kind of analysis can be thought of as an attempt at measurement of a particular construct, and what that construct would be. Take again, the case of the DG: If it is found that women on average tend to donate more relative to men, can we say, then, that women are *more generous* than men? Equally, are students *less generous* than the non-student population given that they typically donate less than other players? Statements like these certainly abound in the literature that discusses these findings, but the interpretation of this type of finding calls for caution, especially against overgeneralization. If women are found to give more on average in the DG, this certainly picks an interesting fact about gender in relation to the game, but the result does not in itself provide enough clues about the ways in which it can be extrapolated to other realms outside of the lab, and this is at least in part due to the fact that that the construct representing the quality or property that is being supposedly measured is itself often only loosely defined, or up for contention.

For differences in results across populations to carry significance or meaning beyond the experimental setting, we would need a clearly defined construct that the game is intended to represent, as well as sufficient background knowledge about the workings of this construct in other settings. In the absence of these, any interpretation must remain local and descriptive: population X tends to give more than population Y *within the game*, or, more cautiously, population X behaves more generously than population Y *in this specific experimental context*.

In sum, the essence of experiments like the DG and UG conducted across different populations rests in the assumption that these games could gauge characteristics of specific groups or communities, and thus, that they *measure* stable properties or enduring qualities of groups or types of people. However, without a strong theoretical stance and background knowledge that tells us what exactly is being *measured* by these games, we are very limited in the way in which we can use effectively this knowledge to infer behaviour outside the lab.

## Is it all about social norms?

Recall once more Engel’s meta-study and his attempt to synthesize the body of evidence from dictator game (DG) experiments to date (Engel, 2011). A particularly noteworthy aspect of this meta-analysis is that it also references a group of highly influential studies from the 2000s showing that the amounts Dictators allocate to Recipients can substantially decrease (or even disappear) under two specific conditions: (1) when Dictators are given the option to conceal their choices, they tend to transfer smaller amounts to Recipients; and (2) when the DG is modified so that Dictators can not only give but also take from Recipients, they frequently choose to do so (Bardsley, 2008; Dana et al., 2006, 2007).

These specific findings hold significant relevance, as they led a group of experimentalists to advocate for a reassessment of was had been until then the conventional interpretation of dictator game (DG) results. This conventional interpretation was well represented, for example by Camerer and Fehr’s dictum whereby “dictator games measure pure altruism” (p. 73).

According to the more critical stance by Bardsley, Dana, and others, the implications of the results of the modified scripts suggested that the DG did not only *not* measure pure altruism (why if so, would Dictators take money from Recipients, when given the chance?) but more generally: the DG was possibly an unsuitable instrument for studying prosocial behaviour. Instead, it could serve at best as a tool to illustrate either the influence of *social pressure* on participants (1) or to reveal *methodological artifacts* (2).

According to the *social pressure* interpretation results should be read as showing the behaviour of participants who were motivated by the anticipation of perceived potential reproach (i.e., they wanted to avoid looking mean or stingy, but they would not have willingly shared their money had they not been under observation). A second group of authors had an even bleaker outlook on the experiment and interpreted its standard results as invalid, i.e., a mere *methodological artifact*: if when given the choice, Dictators sometimes ‘take money from’ rather than ‘give money to’ Recipients, then their seemingly generous behaviour should be interpreted as the result of the *demand effects* of the experimental setting. According to this view, participants that appear generous are merely responding to what they think experimenters expect of them; they just are trying to be “good experimental subjects” (Bardsley, 2008). In addition to these two views, a third group of authors sought to find a way out from the more pessimistic outlook on the DG usefulness for the social sciences. According to this third position, the DG was still an interesting tool to study *sensitivity to different normative scenarios*, thus granting it a similar role to that which Guala attributed to the UG: the game could be used to study how people perceive and understand appropriateness or norms (Jimenez-Buedo & Guala, 2016; Levitt & List, 2007; Zizzo, 2010).

Empirically, too, this led to experimental work that integrated all these results and interpreted them as an instance of norm-following behaviour (Krupka & Weber, 2013). By using an incentivized elicitation method for identifying what players thought was to be expected of them, Krupka and Weber showed that in both cases (when Dictators gave to Recipients in the standard DG, *and* when they took from recipients in the modified DG), their behaviour could be explained as driven by social norms, i.e., dictators were following what they thought was, in each occasion, normatively appropriate behaviour.

Thanks in part to this theoretical reflection, and to this and other sets of empirical studies [notably those inspired by Bicchieri’s account of social norms (2005), such as Bicchieri and Xiao (2009)] the prevailing view today is that both the DG and the UG are normatively laden environments that can serve as useful tools for studying the relationship between social cues, normative contexts, and behaviour. Yet this is not equivalent to claiming that these games *measure* social norms, for a crucial question remains regarding what it would mean, exactly, to *measure* a norm.

The work of Krupka and Weber is instructive in this regard. Their studies do not actually employ the DG to measure social norms. Instead, they use survey questions—monetarily incentivized in Krupka and Weber’s design— to elicit from experimental subjects their beliefs about what they think is *the socially appropriate level of donations in a particular game.* Then these participants’ answers are subsequently related to their observed behaviour in the DG. Krupa and Weber’ findings show a consistent pattern: individuals tend to act in accordance with their beliefs about what constitutes appropriate behaviour. The norms in these experiments are elicited through standard questionnaires that ask about very local, specific beliefs about the appropriateness of subsequent actions in the game, and do not refer to broad normative conceptions regarding rules of fairness or prosocial behaviour in general. Again, the interpretation and meaning of the norm here is local and the measuring of it comes through a standard questionnaire technique (and thus is not directly measured by looking at the behaviour of participants in the DG). Behaviour in the game are then examined to see the coherence between these actions and the beliefs about appropriateness.

While this result (regarding coherence between beliefs and actions) might not be surprising in itself, the experimental evidence from the dictator game together with the participants view on appropriateness allows for a finer-grained examination of subtle variations in normative beliefs and their relationship to behavioural choices. In this sense, such experiments make it possible to observe how changes in perceived social expectations correlate with changes in patterns of giving or sharing.

This line of research has been highly influential, shaping the interests of economists and other empirical social scientists. The currently dominant theoretical framework for understanding social norms is Cristina Bicchieri’s account (2005), which has remained conceptually robust while providing a foundation for a wide range of experimental investigations. Her framework has been applied to norms operating across diverse and often highly context-specific domains, such as understanding cooperation and rule-following in situations involving corruption, tax compliance, or public health behaviours.

Nevertheless, describing these experimental approaches as *measuring* social norms would, once again, be an overstatement. What these studies accomplish, more modestly, yet no less importantly, is a more nuanced understanding of the complex interplay between normative beliefs, behaviour, and preferences. Examining beliefs about norms and relating them to behaviour in the laboratory does not, in itself, amount to measuring norms, at least not through the dictator game (DG). To see this more clearly, consider an analogous example unrelated to norms: collecting data on participants’ political preferences and then comparing these to their behaviour in the DG would not justify the claim that the DG is a tool for measuring political preferences.

There remains, however, one more possible way to defend the claim that experimental games can serve as instruments of *measurement*. This line of argument, to which we turn in the next section, draws on Guala’s proposal that behaviour in the ultimatum game can be used to capture cross-cultural variation in individuals’ *sensitivity to social norms*.

## What did we learn by having the Machiguenga play the ultimatum game? The problem of interpretative holism

Both the ultimatum game (UG) and the dictator game (DG) have been used to investigate various aspects of prosociality and norm sensitivity. We have already argued against the idea that the UG and the DG can act as measuring tools for prosociality or whether they can be meaningfully interpreted as measuring social norms. Still, it remains to be examined whether these games can meaningfully function as “social thermometers” for cross-cultural comparison, the idea being that, because the games are standardized, they could provide a portable means of measuring social behaviour across contexts.

In this section, we turn our attention specifically to the UG in order to address an additional challenge: the comparability of results across different cultural settings. To this end, we examine Joseph Henrich’s implementation of the UG among the Machiguenga, which, as previously noted, figures prominently in Guala’s discussion of the measurement potential of these games. This case also lies at the core of the broader MacArthur Foundation project, which was explicitly inspired by the idea that the standardization of experimental games allows for meaningful cross-cultural comparison.

The prevailing reading of the Machiguenga ultimatum game and its results is that Henrich’s experiment adds to our knowledge about the effect of cultural differences in economic decision-making. According to this interpretation, the results show how the same stimulus (the UG) gives rise to different types of economic behaviour in different contexts (Los Angeles and the Peruvian Amazonia). The UG shows that economic rationality is not universal, but it is instead mediated by cultural traits.

Henrich’s article does indeed show how the Machiguenga players behaved very differently from Western participants (who typically offer something close to a 50/50 split). When playing the UG, the Machiguenga systematically offered much lower amounts (often as low as 20% of the initial allocation). These offers were, in turn, almost never rejected by their counterparts. In Guala’s narrative, the fact that participants from the Peruvian Amazonia behaved differently in front of a standardized game is an argument in favour of the idea that the UG is a *social thermometer measuring norm sensitivity*.

It should be noted that, in his work, Henrich did not limit himself to making the Machiguenga play the UG, but also accompanied his exercise with ethnographic work, some of which came from his own previous anthropological research (Henrich, 1997). In fact, Henrich’s paper is not limited to presenting the empirical results of his experiments, but he also attempts at shedding some light into the reasons why the Machiguenga are outliers in their UG behaviour. Henrich does this by interpreting this behaviour in light of his own ethnographic studies and through post-experimental surveys. Henrich explains how the Machiguenga viewed the UG as a “fun game”: respondents in the game did not interpret low offers as evidence that proposers were “screwing” them, but just as bad luck. Once a subject had been (randomly) assigned the role of respondent, he could only stoically accept that a given proposer would profit from his advantageous position in the game. Henrich also provides context that might explain how they viewed the game as a mere entertaining activity, by describing how the Machiguenga had generally very limited experience with economic interactions outside their closest kin, and that most of their material needs were met within the family.

Let us now turn to the idea that the ultimatum game (UG) serves as a tool for measuring norm sensitivity. Taken at face value, this assertion would imply that low offers (and the ensuing high acceptance rates) should mean a lower sensitivity to norms (and conversely, higher offers would mean a higher score on norm sensitivity). If the UG measures norm sensitivity, an unfortunate implication would be that, when compared to Angelenos, the Machiguenga just score low on this metric: they are less sensitive to the norms embedded in the UG (such as reciprocity, or fairness). But this clearly seems like an undesirable, nonsensical conclusion. Instead, a much more plausible story to be told in view of Henrich’s fascinating exercise is to underline (and give the prominent role this fact deserves) that the way that the Machiguenga interpret and play the UG is just different from the way in which the subjects in Los Angeles do so. Another way to put this, a more radical one, is that the Machiguenga played a *different game altogether* than participants in Los Angeles, even when given the same *standardized* script. For them, the UG embodied different norms than for Angelenos, rather than the Machiguenga being insensitive to the norms the UG embodies. It is easy to see how under this interpretation, the hope of measurement vanishes: different results in different locations would not mean different measurements of the same variable, nor of the same construct. The results instead simply empirically show the differences in the way different human groups play, demonstrating that the game means different things for different communities. This is an interesting finding in its own right, but a measurement it is not.

The example thus shows that despite standardization of the game itself,^7^ the UG script, understood in context, meant something else to the Machiguenga than it did to participants in Los Angeles. Anthropologists refer to this type of phenomenon as *interpretative or hermeneutic holism*, whereby the meaning of a particular cultural practice or trait cannot be understood in isolation but instead takes its meaning from the whole background of cultural practices within a community (Risjord, 2007). *Interpretative holism* poses a problem for the translation of singular traits or institutions to other languages, or to other, outside, observers. According to interpretative holism, this is why the *potlatch*, the *kula*, or child marriage do not make sense outside context, or rather, make a different sense altogether. We cannot implement the UG in very different contexts and expect that its meaning translates easily across cultures: the UG may be played with the same protocols in very different contexts, but its meaning will likely be different, and local.

A few observations are thus in order. First, an obvious one: if the UG cannot be assumed to measure norm sensitivity, then the Machiguenga are surely as sensitive as Angelenos to some social norms (notably, their own norms). Second: the evidence points to the fact that the UG embodies different norms for different people, and we cannot be sure ex ante of what is the norm that the ultimatum game embodies for a particular group (in this case, the Machiguenga) but can only find it out through careful ethnographic work involving interviews, participant observation and the like. This has implications for our ability to interpret the UG results and it also determines what we can and cannot learn from them.

Playing the UG in different cultural contexts allows us to learn how participants in diverse settings interpret (and engage with) this experimental script. However, what we learn from such studies about prosociality or norm sensitivity is far from straightforward. By inviting Machiguenga men to play the UG, Henrich’s study revealed fascinating insights about the Machiguenga and about human behavioural variability more broadly. Yet it is important not to overlook that much of this understanding stemmed from careful ethnographic investigation, rather than from the numerical outcomes produced by a superficially standardized “social thermometer.”

## Conclusion: constructs, standardization, and the problem of coordination

According to the received view, one of the reasons why games like the DG or the UG are helpful to explore ‘human sociality’ is that “the situation can be tightly controlled, because the simple game can be played with all classes of participants and, of course, because decisions are incentivised” (Engel, 2011).

This widely shared position (also articulated by Guala) maintains that certain features of these games, like the use of a clear, controlled script and the use of economic incentives, promotes experimental control and standardization.

The Machiguenga case, despite having been cited to support the concept of a ‘social thermometer’, highlights a crucial limitation: while we may achieve standardization in the mechanics of games like the ultimatum game (UG) and the dictator game (DG), this standardization remains only surface-level. We can standardize the mechanics of the game, understood as a material intervention based on a standard script, but this standardization does not grant uniformity in what the game signifies to different participants. Because these games may represent different things to different subjects, standardizing the mechanics of play does not guarantee consistency in participants’ interpretations. Yet, for the DG and UG to function as “thermometers” of sociality, their interpretation must be uniform across individuals, groups, and experimental arms.

Consequently, despite procedural standardization, these games fail to resolve the problem of *measurement standardization* as conceived in the new philosophy of measurement. This problem concerns how to compare results from tests that inherently vary within and between individuals—a process that requires the prior establishment and maintenance of consistent conceptual ascriptions or agreed-upon reference units and practices (Barwich & Chang, 2015).

In the case of the DG and UG, we do not observe this kind of collective harmonisation. On the contrary, as the literature shows, the practices surrounding their implementation are diverse, and researchers often interpret the same experimental outcomes as evidence for different constructs, such as generosity, altruism, fairness, or norm-following. As we have seen, different researchers use these scripts to test varied hypotheses about conceptually distinct phenomena. But even when experimenters may claim to measure a particular construct, the interpretation of the findings typically remains local, given that there are no obvious counterparts or correspondences between what goes on in these experiments and phenomena outside of the lab.

So what explains the many references to *measurement* by practitioners and commentators, then? Perhaps it has to do with the fact that these games generate numerical outcomes under conditions that seem standardized (in the above mentioned sense). The experimental results *appear* to constitute measurement in part because they do produce numbers. However, these numbers have only local significance. Without addressing the underlying problem of coordination, there is no genuine measurement, even if numerical data are produced.

As we already mentioned, the *problem of coordination* deals with “the way in which knowledge of how the instrument works links the operational level with the theory of the phenomenon in a meaningful way” (Barwich & Chang, 2015, p. 209). This problem thus refers to the challenge of establishing a reliable and consistent link between a theoretical concept (e.g., temperature, well-being, or fairness) and the empirical operations or instruments used to measure it.

In contrast, the interpretation of the DG and UG is inherently local precisely because the constructs they are meant to represent often remain undefined, or only provisionally posited. These constructs can shift, and as a result, the numerical outputs cannot be meaningfully compared across studies or populations. While such experiments do test hypotheses about prosociality, altruism, or norm compliance, they do so without committing to clearly specified theoretical constructs. Much of the research conducted with the DG and UG is therefore exploratory rather than genuinely measuring. It produces numbers without resolving the conceptual and coordinative challenges that make measurement meaningful. These exercises cannot answer thus the central question of whether an instrument or procedure truly measures what it is intended to measure, precisely because that purpose is often left open ended.

One could still try to defend the view that despite these limitations, we may still be in the nascent stages of some collective *measuring endeavor* that will in time yield its fruit. According to this view, the currently extensive body of DG or UG experimentation would eventually converge on a unified, well-defined construct (broadly related to concepts such as prosociality, altruism, generosity and the like). However, the current imbalance between the vast amount of empirical evidence and the sparse theoretical discussion around constructs in the DG and UG experimentation is a critical issue that should temper the optimist view whereby we are perhaps in the early stages of some collective effort in measurement. This disparity between theoretical and empirical efforts places DG and UG experimentation closer to exploratory experimentation and away from a systematic, coordinated measurement endeavor, and raises doubts about whether these scripts can serve as a tool in an inductive process that ultimately converges on a stable and broadly agreed-upon construct around the notion of prosociality or social preferences.

The UG and the DG deal with behaviour that surely has to do with these and related notions (whether altruism, inequality aversion, norm following or social pressure) but this does not mean that these games *measure* any of these constructs and there is little evidence of collective efforts at arriving, iteratively, at theoretical refinement or conceptual clarification of these phenomena via measurement: there is often no shared conceptual framework guiding the meaning of the constructs involved.

This should by no means pre-empt all social scientific interest in these scripts. The weak link of the dictator game and the ultimatum game to specific constructs does not invalidate their use in social scientific experimentation. These games remain scientifically useful for purposes other than measurement: they serve as props that elicit diverse responses from participants and can allow for varied hypothesis testing. They remain, for their sensitivity to subtle environmental changes, versatile tools for studying human behaviour in controlled settings.

However, it is precisely because of these characteristics—the games’ ability to evoke varied responses and their sensitivity to subtle environmental cues—that they are less than ideal measurement devices. While the UG and the DG excel at producing intriguing results, our current uses of them lack the standardization and coordination required for rigorous measurement and comparison across different contexts or individuals.

## Data Availability

Not applicable.

## Footnotes

1 In the DG, the experimenter allocates some fixed quantity of money to player 1, the Dictator, who then must decide how much, if any, he wants to share with player 2, the Respondent. The game ends there and Dictator and Recipient collect their share of the initially endowed sum according to the Dictator’s offer. The results of the standard DG show that roughly half of the Dictators depart from the earnings maximizing strategy and choose to give some money, the mean allocation being 20% of the initial endowment. Moreover, a consistent minority of dictators choose to split the sum in two similar sizes (Camerer, 2003). The UG has a bit more structure that the DG (the DG can actually be understood as simpler, degenerate version of the DG): the ultimatum game typically involves two players. Player A or Proposer is given a sum of money by the experimenter and has to propose how to divide it between the two players. Player B or Respondent can either accept or reject the proposed division. If accepted, the money is split as proposed. If rejected, neither player receives anything. The standard results show that proposers often offer a relatively fair split, typically around 50% of the total amount, to avoid rejection by responders and ensure the offer is perceived as fair. Responders frequently reject offers they perceive as unfair, even if it means receiving nothing. These results, first found by Güth et al. (1982) have been replicated in numerous studies across different contexts (e.g., Bolton & Zwick, 1995; Camerer & Thaler, 1995).

2 The number of references that take either game as a measurement tool is immense. For a discussion and some experimental evidence of which variants of the DG can be considered a more accurate measure of altruistic preferences, see Grossman et al. (2020). For an example of a use of the UG interpreted as measurement in a very different context, see Torta et al. (2013), using the UG as a measurement tool for anthropomorphism in human–robot interactions.

3 Here, we use construct in the sense that it takes in a Campbellian framework (Shadish et al., 2002), where a construct is a theoretical concept that is not directly observable or measurable, such as intelligence, social anxiety, or fairness. Accordingly, construct validity refers to the extent to which the inferences about the underlying theoretical constructs or concepts the investigator intends to assess are accurately captured by the specific operationalizations in a given study.

4 Both Hasok Chang and Eran Tal view measurement as an epistemically iterative and self-correcting process. Chang adopts a constructivist–operationalist stance, seeing measurement as the historical refinement of practices and instruments. Tal shares this view but frames it as model-based, portraying measurement as iterative model-mediated representation.

5 In other cases, that are equivalent in structure, the two arms of the experiment have recipients that are identified by an equivalent marker. For example, in the baseline treatment the recipient is from the Dictator’s same ethnic group and in the treatment, the recipient is from a different ethnicity (see Whitt & Wilson, 2007).

6 As noted by one of the referees, some very broad notions of prosociality may classify ethnic bias as falling within what we can consider prosocial behaviour. Here, however, we adopt a narrower and more conventional definition, restricting prosociality to behaviours intended to benefit others and excluding in-group bias and in particular, behaviour that involves negative treatment of out-group.

7 Though Henrich’s article precedes the project of the MacArthur Foundation, it can be considered part of the project and shares with the endeavour a meticulous effort for the standardization of the experimental procedures: Henrich and the rest of the team went through great care to ensure that participants fully understood the nature of the game being played and often, this implied that the researchers had to do several adjustments to the game (like the type of token they would then exchange for money after the game is played, etc.). For example, in the case of the Machiguenga, and given the social structure in this community, the game was only played by the men in the community.
